# Elastography in the Urological Practice: Urinary and Male Genital Tract, Prostate Excluded—Review

**DOI:** 10.3390/diagnostics12071727

**Published:** 2022-07-16

**Authors:** Vasile Simon, Sorin Marian Dudea, Nicolae Crisan, Vasile Dan Stanca, Marina Dudea-Simon, Iulia Andras, Zoltan Attila Mihaly, Ioan Coman

**Affiliations:** 1 Urology Department, “Iuliu Hațieganu” University of Medicine and Pharmacy, 400347 Cluj-Napoca, Romania; simonvasile89@yahoo.com (V.S.); drnicolaecrisan@gmail.com (N.C.); vasilestanca@yahoo.com (V.D.S.); dr.iuliaandras@gmail.com (I.A.); jcoman@yahoo.com (I.C.); 2 Municipal Clinical Hospital, Tabacarilor 11, 400139 Cluj-Napoca, Romania; mzottiko@yahoo.com; 3 Radiology Department, “Iuliu Hațieganu” University of Medicine and Pharmacy, Clinicilor 3–5, 400347 Cluj-Napoca, Romania; 4 Obstetrics and Gynecology Clinic II, “Iuliu Hațieganu” University of Medicine and Pharmacy, 400124 Cluj-Napoca, Romania; marina.dudea@gmail.com

**Keywords:** elastography, renal, testicular, penile, urethral, urethral sphincter, urinary bladder

## Abstract

The aim of this article is to review the utility of elastography in the day-to-day clinical practice of the urologist. An electronic database search was performed on PubMed and Cochrane Library with a date range between January 2000 and December 2021. The search yielded 94 articles that passed the inclusion and exclusion criteria. The articles were reviewed and discussed by organ, pathology and according to the physical principle underlying the elastographic method. Elastography was used in the study of normal organs, tumoral masses, chronic upper and lower urinary tract obstructive diseases, dysfunctions of the lower urinary tract and the male reproductive system, and as a pre- and post-treatment monitoring tool. Elastography has numerous applications in urology, but due to a lack of standardization in the methodology and equipment, further studies are required.

## 1. Introduction

Elastography is a non-invasive imaging technique complementary to conventional ultrasound that determines the stiffness of the analyzed organ or lesion. According to the physical principle underlying the method, elastography is divided into real-time elastography or strain elastography (RTE) and shear wave elastography (SWE) with its two subtypes: bidimensional SWE (2D SWE) and point SWE (pSWE), also known as acoustic radiation force impulse (ARFI) [1].

This technique is already used to evaluate a wide range of pathological conditions, including breast, liver, thyroid and uterine cervix disease [2,3,4,5,6].

In urology, elastography has a widely studied and proven role in evaluating renal transplant, prostatic and nephrological diseases [7,8,9]. These topics were not included in the review.

This paper aims to review the less common elastography applications in urological practice.

## 2. Materials and Methods

An electronic database search was performed on PubMed and Cochrane Library. Papers published between January 2000 and December 2021 were retrieved, using the following search terms: kidney/renal elastography”, ”testicle/testicular elastography”, “penis/penile elastography”, “urethra/urethral elastography”, “bladder elastography”, “urethral sphincter elastography”.

Inclusion criteria:

- Full text available in English
- Available patient data
- Human related studies
- Non-experimental studies, in-vivo

Exclusion criteria:

- Nephrological disease
- Transplanted kidney
- Prostate evaluation
- Articles not meeting the inclusion criteria

The database search yielded a total of 1197 articles. After applying the inclusion and exclusion criteria, 94 papers were retained.

**Figure 1:**
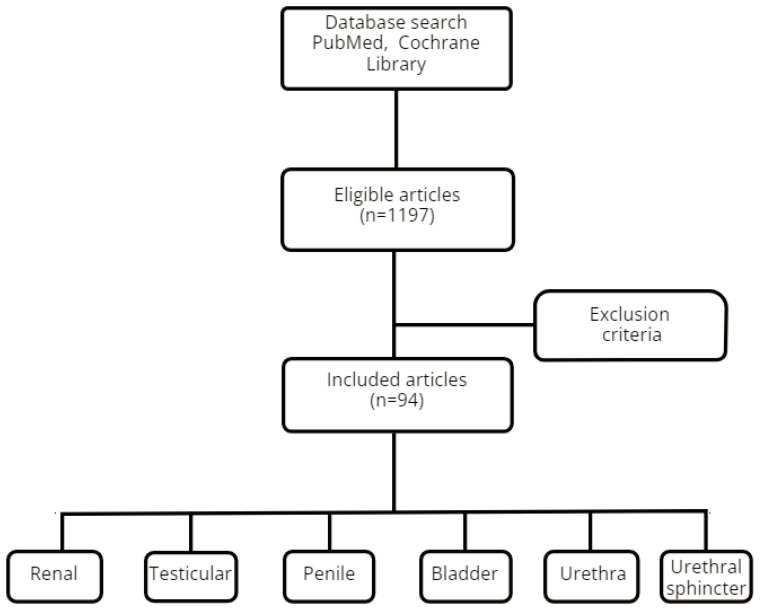
STARD diagram of the inclusion and exclusion criteria.

The articles were grouped and discussed by organ, method and pathology as presented in Figure 1.

## 3. Results

### 3.1. Kidney

#### 3.1.1. Normal Values for Renal Stiffness

##### Point Shear Wave Elastography (pSWE or ARFI)

*Age dependance.* The values measured in newborns and infants were lower (mean 1.7 m/s, range 0.8–2.5 m/s) than the ones determined in a broader age group (0–20 years) where values of 2.10 ± 0.43 m/s (ventrolateral) and 2.30 ± 0.37 m/s (dorsal) were measured [10,11]. Contradicting correlations between the pSWE values and age were found, depending on the study [11,12,13,14].

*Gender dependence.* No direct correlation between gender and the pSWE values was found. Only after applying multivariate linear regression analyses was a correlation between gender and the pSWE values detected, with slightly higher values for women [14].

*Cortex* vs. *medulla*. In a small study on volunteers with superficial kidneys, cortical stiffness values were higher than the medulla ones: 15.4 ± 2.5 kPa vs. 10.8 ± 2.7 kPa, respectively [15].

*Manufacturer.* The pSW speed measured in the cortex varied depending on the device’s manufacturer, ranging from 1.49 to 2.82 m/s [12,14,16].

##### 2D SWE

The normal values as determined by 2D SWE were 5.0 ± 2.9 kPa in a study on 127 healthy volunteers [17]. Similar values—4.7 ± 1.7 kPa for the left renal cortex and 4.5 ± 1.5 kPa for the right—were found by Cai et al. in the normal contralateral kidney in 117 patients with renal masses [18].

At the time of this review’s compilation, there were no available studies on 2D SWE value differences according to age, gender, manufacturer or anatomic renal component.

**Conclusion:** Further studies on the influence of age and US manufacturers on pSWE values are necessary.

**Figure 2:**
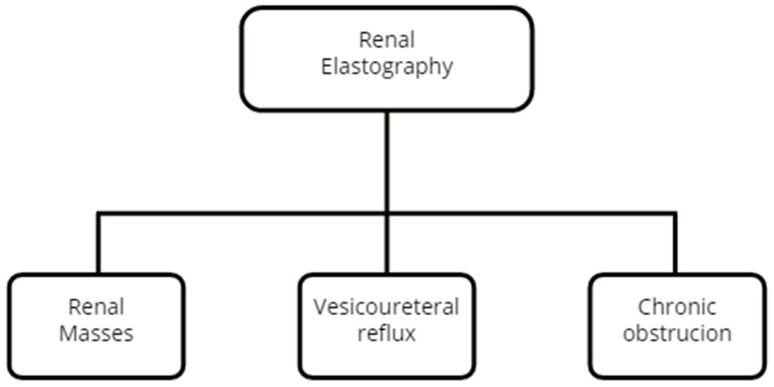
Renal pathologies assessed by elastography.

The published usage of renal elastography is summarized in Figure 2.

#### 3.1.2. Renal Masses

##### pSWE

The ability of pSWE to differentiate renal masses represents the focus of several papers. A study on 44 patients predefining its groups as renal cell carcinoma (RCC), angiomyolipoma (AML) and pseudotumor concluded that pSWE failed to distinguish between RCC and AML but was able to differentiate both AML and RCC from pseudotumors. Pseudotumors in this study were defined as anatomical variations of the normal kidney that can mimic a renal tumor (hypertrophied column of Bertin and duplex kidney) [19].

Another study evaluated the usefulness of combining pSWE and machine learning as a diagnostic technique. The results obtained support the conclusion of the previous study, as pSWE cannot differentiate between RCC and AML when only the stiffness of the tumor is measured. However, combining the velocities of the tumoral mass, normal cortex and medulla using logistic regression in the machine-learning process showed a statistical difference between RCC and AML. These results support the idea that a malignant tumor may alter the normal surrounding tissue, and this can be used to differentiate the nature of renal masses [20].

A study conducted by Goya et al. that compared the elasticity of malignant, benign and infectious masses using pSWE showed a statistical difference (*p* = 0.033) between malignant and benign masses but overlapping results compared to infectious masses. This overlap can be attributed to the evolutive stage of the abscess undergoing swelling, induration or liquefaction [21].

Thaiss et al. used pSWE to study computed-tomography (CT)-indeterminate renal masses. This study measured the pSWE and shear wave ratio (SWR). The computed SWR was the ratio between the region of interest’s values of the lesion and the values of the surrounding tissue. Clear cell (ccRCC), papillary (pRCC), chromophobe renal carcinomas (chRCC) and oncocytomas had different stiffnesses and shear wave ratios. Point SWE could differentiate between three histopathological entities (ccRCC, 3.4 ± 0.8 m/s; pRCC, 2.2 ± 1 m/s; chRCC, 1.9 ± 0.5 m/s). Oncocytomas showed higher shear wave ratios than papillary and chromophobe tumors but could not be differentiated from ccRCC [22].

**Conclusion:** Inflammatory masses are a confounding factor for pSWE evaluation due to their relatively fast evolutive characteristics. Although promising, the study by Thaiss et al. regarding the use of pSWE in the differentiation of RCC types needs further validation.

To the best of our knowledge, no studies have used pSWE to differentiate RCC and transitional cell carcinomas (TCCs).

##### 2D SWE

Two studies described the ability of 2D SWE to differentiate RCC and AML, with contradicting results on which of the two entities has the higher stiffness. The main reason for this disparity seems to be the size of the masses included in the study, as one research included only tumors < 4 cm with a mean of 2.96 ± 0.89 cm, while the other enlisted patients indiscriminately, with a mean diameter of 5.23 ± 3.42 cm [18,23].

**Conclusion:** There is a need for further studies that would stratify the tumoral masses by size for 2D SWE to differentiate between RCC and AML.

There are no current studies on 2D SWE for the differentiation between RCC subtypes and between RCC and TCC.

##### RTE

Strain elastography using the strain ratio (SR) in renal masses has been used to differentiate RCC from AML. In two studies, the SR was calculated by placing the first region of interest (ROI) on the tumor and the second on the normal renal cortex. The strain ratios for RCC and AML were 3.4 ± 0.3 vs. 1.1 ± 0.1 and 4.05 ± 2.17 vs. 1.43 ± 0.94, respectively. Both studies noted significant statistical differences when comparing these two entities [24,25].

Another study conducted by Tan et al. used an inverted position of the ROIs, resulting in an SR of 0.64 ± 0.15 for RCC and 0.15 ± 0.06 for AML. The differences were also statistically significant [26].

When considering the size of the lesion, the SR varied for both RCC and AML. Strain elastography differentiated between these two entities when limits of <20 mm, 20–40 mm and >40 mm were used to stratify the lesions [24,26].

When comparing RCC and transitional cell carcinomas (TCCs), the SR values were 5.18 ± 1.12 for TCC and 4.04 ± 0.72 for RCC, respectively, with statistical differences [27]. Oppositely, Onur et al. showed lower strain ratios for TCC compared to RCC. When interpreting these findings, the small number of TCC patients (*n* = 4) should be considered [25].

**Conclusion:** There are no studies that evaluate the different RCC subtypes using RTE.

#### 3.1.3. Vesicoureteral Reflux

##### pSWE

When studying the effect of vesicoureteral reflux on the kidney in a pediatric population, Goya et al. found negative correlations between pSW values and vesicoureteral reflux grade and the DMSA scintigraphy scarring score. Kidneys with normal DMSA scintigraphy had mean pSW values of 2.39 ± 0.23 m/s vs. 1.83 ± 0.44 m/s for kidneys with abnormal DMSA scintigraphy [28].

##### 2D SWE

Kalyoncu et al. reached the same conclusion as the paper mentioned above regarding the relationship between stiffness values and kidney with scar formation. However, they found no difference in the SWV values if no scarring tissue was present and it was a normal contralateral kidney [29].

##### RTE

To the best of our knowledge, a single study used strain elastography in vesicoureteral reflux in the pediatric population. Karabulut et al. found no elastosonographic data difference in the strain ratio between renal units with and without a differential function decrease and between renal units with and without scar formation [30].

**Conclusion:** Further studies are necessary to establish if RTE truly cannot differentiate between renal units with normal and decreased differential function in the case of vesicoureteral reflux.

#### 3.1.4. Ureteropelvic Junction Obstruction (UPJO)

##### pSWE

In a study on 51 children that compared pSWE in normal vs. hydronephrotic kidneys, 19 patients had hydronephrosis and UPJO was the cause of obstruction in 7 cases. The SW velocities were higher in hydronephrotic kidneys compared to normal kidneys, but there was no statistical difference between hydronephrotic kidneys with and without UPJO [31].

##### 2D SWE

2D SWE showed conclusive results in a study on postoperative follow-up on 31 pediatric patients with the patients that underwent standard open dismembered pyeloplasty by the same surgeon having lower SW velocities at three months (2.73 m/s) and six months (2.57 m/s) compared to the preoperative mean reference value (3.21 m/s). The SW velocities remained unchanged or increased in patients who did not undergo surgery [32].

**Conclusion:** The correlation between the hydronephrosis grade stratification and the pSW velocities was not studied. The few published papers suggest a still-to-be-explained discrepancy between the pSWE velocity values of the kidneys in patients with hydronephrosis due to vesicoureteral reflux vs. patients with hydronephrosis due to UPJO.

2D SWE may be useful to monitor the patients’ evolution after corrective surgery for UPJO.

## 3.2. Testicle

### 3.2.1. Testicle Normal Values

#### pSWE

In a study conducted on 66 patients, the mean SW velocity values were similar for the upper and lower testicular poles (1.15 m/s) and lower for the center of the testis (0.9 m/s) [33].

In a study on 130 normal patients compared with patients with testicular microlithiasis and testicular masses, the SW velocity values of normal testicles were statistically significantly lower (0.76 m/s (95%CI: 0.75–0.78)) than the pathological groups [34].

#### 2D SWE

Examinations of 110 healthy volunteers revealed the central region yielding the lowest SW values (3.14 ± 0.35 kPa), followed by the upper and lower poles (3.94 ± 0.90 kPa; 3.94 ± 0.97 kPa). The measurement in the poles included the testicular capsules, thus explaining the increase in stiffness. Differences were found when the stiffness of the posterior and anterior capsules was measured (5.96 ± 1.46 kPa vs. 6.27 ± 1.58 kPa) [35]. The authors do not define how they included the testicular capsule in the ROI. The inclusion of fibrous peritesticular tissue is not relevant for the condition of the testicular parenchyma. Furthermore, the inclusion of fibrous tissue close to the transducer leads, as expected, to an increase in rigidity.

#### 2D SWE

3D SWE did not show statistical differences between different regions of the testicle [36]. In a pediatric population of 52 boys aged 1–92 months, testicular stiffness values were negatively correlated with age and weight [37].

**Conclusion:** There are few studies aimed only at describing normal testicular stiffness. As expected, the pediatric population’s stiffness decreases with increasing age and weight. However, there are no studies on the age dependence of testicular stiffness in the adult population to the best of our knowledge. Methodological and machine-associated variances should also be addressed.

**Figure 3:**
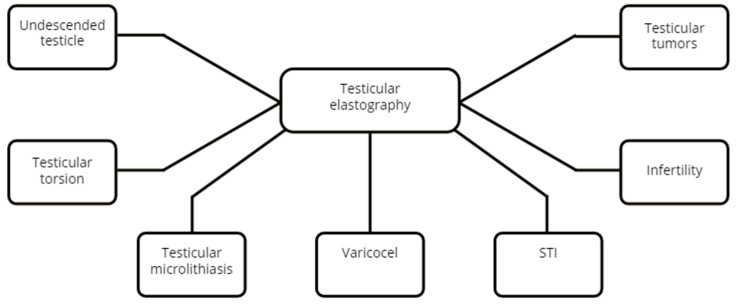
Testicular pathologies assessed by elastography.

The published usage of testicular elastography is summarized in Figure 3.

### 3.2.2. Undescended Testicle(UDT)

#### pSWE

pSWE was used to describe the testicular SW velocity of pediatric patients after orchidopexy with 0.75–2.8 (median, 1.1) m/s for the operated group vs. 0.62–1.2 (median, 0.84) m/s for the contralateral testicle and 0.65–1 (median, 0.82) m/s for the control group [38].

#### 2D SWE

In a study on 29 patients with a mean age of 7.52 years, Ucar et al. concluded that there was a significant difference between undescended testes and contralateral descended testes in terms of SWE values (9.6 ± 3.15 kPa vs. 4.76 ± 1.5 kPa) but no difference in volume [39].

In a retrospective study including 25 UDT vs. 54 normal testicles in a pediatric population under 60 months, the SWE values in the UDT group had a positive correlation with age and a negative correlation with volume. The opposite was described in the normal testes group [40]. Turna et al. showed elevated stiffness and velocity values not only in the cases of undescended testicles (13.80 ± 4.14 kPa, 2.14 ± 0.29 m/s) but also for retractile testicles (9.64 ± 3.71 kPa, 1.75 ± 0.35 m/s) compared to normal testicles (7.44 ± 2.11 kPa, 1.57 ± 0.21 m/s) [41].

#### RTE

**RTE** can be used as a complementary tool to grayscale ultrasonography to confirm the presence of undescended testicles located in the abdominal cavity or inguinal canal or to establish the persistence of areas of increased stiffness after orchidopexy [42].

**Conclusion:** Both pSWE and 2D SWE can be used to evaluate the elasticity of the UDT before and after surgery. 2D SWE has shown the ability to differentiate between an orthotopic testicle and a retractile one independent of the timing of the examination. No current studies use RTE to describe the UDT other than as just a complementary tool to bidimensional ultrasonography. There is no published information on any potential role of the strain ratio.

### 3.2.3. Testicular Torsion

#### 2D SWE

Sun et al. showed that SWE can be used to establish the diagnosis of testicular torsion. Although this study had a limited number of patients, there was a clear statistical difference between the SWE values in the case of testicular torsion vs. normal testicles (Emean, 78.07 ± 9.01 kPa vs. 22.0 ± 5.10 kPa; Emax, 94.07 ± 6.53 kPa vs. 27.87 ± 5.78 kPa; Emin, 60.73 ± 7.84 kPa vs. 18.90 ± 4.39 kPa; SD, 7.67 ± 0.60 kPa vs. 2.30 ± 0.36 kPa, (*p* < 0.05)) [43].

The ability to correctly differentiate between testicular torsion and acute orchitis has always been a clinical challenge in the urological practice. Xue et al. observed higher SWE values for the testicular capsule (138.76 ± 58.27 vs. 16.40 ± 4.71 kPa, *p* = 0.0001) and twisted spermatic segment (166.61 ± 60.07 vs. 14.14 ± 4.93 kPa, *p* = 0.0001) in the torsion group and the orchitis group. They found no statistically significant difference between groups for the middle parenchyma of the testis (*p* = 0.053) [44].

**Conclusion:** As suggested by the above studies, 2D SWE may be used to differentiate between testicular torsion, acute orchitis and the normal testicle. The accessibility and time spent to reach an ultrasound machine and an examiner able to properly perform testicular elastography should be taken into consideration in the case of testicular torsion. Delays in surgical exploration and spermatic cord detorsion can lead to testicular necrosis, atrophy or loss of function.

No papers on using pSWE and RTE to evaluate testicular torsion were published, to the best of our knowledge.

### 3.2.4. Testicular Microlithiasis

#### pSWE

Few articles describe the use of sonoelastography in diagnosing testicular microlithiasis (TML). Pedersen et al. found no difference between pSWE values in normal testicles and testicles with microlithiasis (0.82 ± 0.127 m/s) [45]. In a later study with a larger number of patients, the same researcher found a slight but statistically significant SW velocity difference in men with TML compared to normal testicles, with higher velocities in the TML group [34].

In another study, Aslan et al. found higher mean pSWE velocities in the pediatric population of 25 patients with TML (1.18 ± 0.22 m/s) compared to the healthy control group of 24 patients (0.88 ± 0.11 m/s). The TML and control group were 6.7 ± 3.17 and 7.9 ± 4.18 years, respectively [46].

**Conclusion:** Due to the low number of patients enrolled in the studies, further exploration of the utility of pSWE in the evaluation of TML is necessary. To our knowledge, no studies used 2D SWE or RTE to evaluate testicular microlithiasis.

### 3.2.5. Varicocele

#### pSWE

Dede et al. used pSWE to evaluate 30 infertile patients with varicocele and found mean SWV of 0.82 ± 0.08 m/s and 0.87 ± 0.09 m/s for the varicocele-bearing testis and normal testis, respectively. The difference was statistically significant. They also found a statistically significant negative correlation between the FSH values and varicocele grade and the elasticity of testes (this means a positive correlation between FSH values and testicular stiffness). The main limitation of this study was the enrollment of only patients with mild oligospermia (10 to 15 million) and no patients with oligoasthenospermia or azoospermia [47].

**Conclusion:** To confirm the utility of pSWE in the evaluation of varicocele, more studies with a larger variety of spermogram parameters are required.

#### 2D SWE

Jedrzejewski et al. showed SW values of 2.5 ± 0.49 kPa in testes with grade I of varicocele, 2.59 ± 0.81 in grade II and 2.80 ± 0.72 kPa in grade III. In contralateral testes, the values were, respectively: grade I—2.39 ± 0.49 kPa; grade II—2.41 ± 0.61 kPa; and grade III—2.42 ±0.85 kPa. An important observation highlighted in this study is that the SWE values were statistically significant only if the difference in testicular volume was >20% [48].

Contrarily, Rocher et al. observed that varicocele-bearing testes were softer than normal ones (2.2 (1.8–2.6) kPa vs. 2.4 (2–2.9) kPa). No significant statistical difference was found between the varicocele-bearing and contralateral testes [49].

Ryu YJ noted no difference in the Emean SWE values during resting states but increased testicular stiffness during the Valsalva maneuver for grade II and III varicoceles [50].

A study conducted by Abdelwahab et al. monitored patients with varicocele before and after surgery and established a cut-off value of 4.5 kPa for postoperative improvement of spermogram parameters. SWE showed a significant negative correlation between the SWE stiffness index and sperm count (million/mL) and total motility, but no significant statistical correlation between SWE values and the percentage of normal-form sperm cells [51].

Turna et al. used SWE to evaluate patients with left varicocele and compared the SWV values in subjects with normal and oligospermia. The varicocele patients with oligospermia had higher SWE values than the normozoospermic patients (6.15 ± 1.96 kPa vs. 4.77 ± 1.16 kPa). All the patients with varicocele showed higher SWE values than the healthy control group (3.79 ± 0.94 kPa) [52].

Erdogan et al. observed higher SW values and velocities in the varicocele-bearing testis and the contralateral testis than in the control group. This study found no correlation between the testicular volume and SW values [53].

**Conclusion:** 2D SWE seems to perform better when there is a significant difference in testicular volume and high-grade varicoceles are examined. 2D SWE values negatively correlated with sperm count and may be used as an alternative method to the spermogram. 2D SWE might be used to predict the postoperative improvement of spermogram parameters after the varicocele surgical cure.

#### RTE

Strain elastography can also be used to evaluate varicocele-bearing testis. Higher strain ratios were obtained when comparing the varicocele-bearing testis with the contralateral testis, indicating that the stiffness was increased in patients affected by varicocele [54,55,56]. Strong inverse correlations were shown between strain elastography and sperm parameters such as total motile count, sperm concentration and sperm normal morphology. Positive correlations were identified between varicocele grade, strain ratios and elasticity scores [55,56].

**Conclusion:** The varicocele-bearing testis is stiffer than the normal testis. Negative correlations have been found between testicular rigidity and sperm parameters.

### 3.2.6. Segmental Testicular Infarction (STI)

Due to the rarity of this pathology, there are only two case reports on elastography of segmental testicular infarction and a small series report on six patients.

In a small series report, six patients with suspected segmental testicular infarction were examined by RT or SWE with no clear conclusion. Surgical exploration and testicular sparing surgery were performed in five out of six cases; thus, no follow-up was available [57].

SWE was performed on a 35-year-old male for whom Doppler US and MRI confirmed segmental testicular infarction. The examinations showed soft areas in color mode in the central portion of the affected area with a mean elasticity value of 1.7 kPa compared to the normal testicular parenchyma, having a value of 2.6 kPa. An SWE re-evaluation was performed ten days after the initial presentation showing an increased stiffness of 22.3 kPa in the same area, denoting the evolutive nature of the pathology [58].

**Figure 4:**
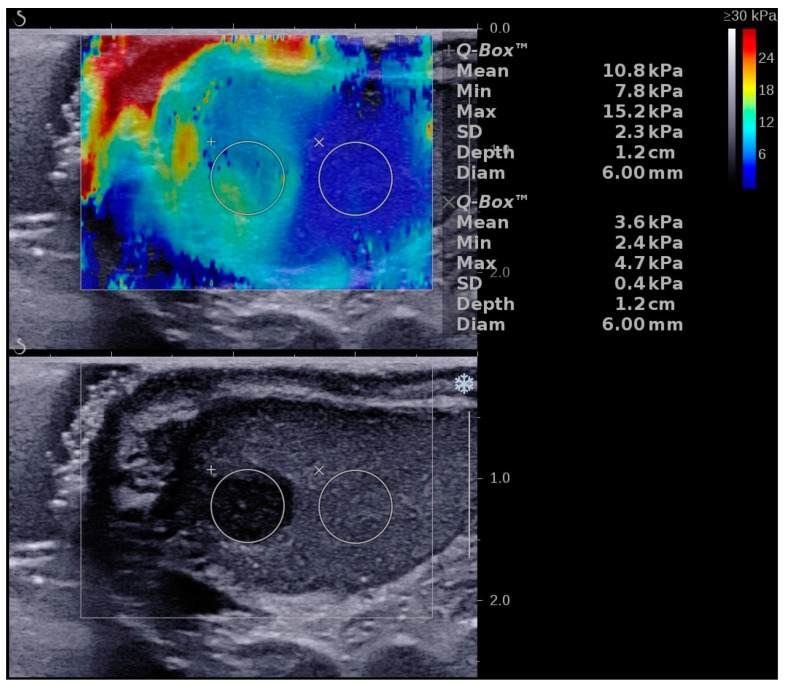
Illustration of a two-dimensional shear wave elastography (2D SWE) performed in a patient with a segmental testicular infarction using the SuperSonic Imagine Aixplorer Ultrasound device. A color scale map is displayed in the upper part of the image, low stiffness is color-coded with blue, while red signifies high stiffness. Numeric 2D SWE results (expressed in kPa) are displayed on the right side of the image. The Q-Box displays the mean, median, minimum, maximum and standard deviation (SD) of the measurements, along with the depth, the diameter of the region of interest (ROI) and the stability index (SI).

RTE was used to evaluate a case of a 33-year-old male with acute testicular pain on the third day of onset. Segmental testicular infarction was diagnosed by Doppler US and CEUS. The initial evaluation showed that the lesion was “soft” and had a strain ratio of 1.14. On further follow-up at 7 and 14 days, the lesion showed a strain ratio of 1.14 and 1.22, respectively [59]. Figure 4 shows different stiffness values in the same STI lession.

**Conclusion:** Due to the fast evolutive nature of this pathology, serial examinations are required to document it properly. The scarcity of such cases impedes more extensive further studies.

No studies that use pSWE in segmental testicular infarction were found.

### 3.2.7. Infertility

#### pSWE

When comparing the pSWE values of 90 infertile men to those of 10 healthy subjects, Yavuz et al. obtained SWE cut-off values distinguishing normal vs. azoospermia (1.465 m/s) and oligozoospermia (1.328 m/s), and oligozoospermia from azoospermia (1.528 m/s), but with low specificity and sensitivity. Significant negative correlations were found between patients’ mean testicular SW velocities and their sperm counts [60].

**Conclusion:** Due to low specificity and sensitivity, pSWE cannot be considered an alternative to semen analysis. It might be helpful as a screening and follow-up tool in male infertility.

#### 2D SWE

SWE was used to correlate various parameters in infertile men. The SWE absolute values varied by cause of infertility, total sperm count and the machine used to perform the examination. In men with oligoasthenoteratozoospermia (OAT), Illiano et al. noted a higher mean SWE value in both testicles when compared to men with normal semen parameters (left testicular stiffness 21.4 ± 5.4 kPa vs. 9.9 ± 1.6 kPa, *p* < 0.0001; right testicular stiffness 22.9 ± 4.8 kPa vs. 9.5 ± 2.4 kPa, *p* < 0.0001). The same study showed negative correlations between the testicular stiffness and total sperm count, concentration and progressive motility [61].

In 91 patients with azoospermia, Hu et al. managed to discriminate between patients with normal spermatogenesis and hypospermatogenesis via a biopsy using SWE with a cut-off value of 1.55 kPa [62].

Erdoğan et al. examined 100 testicles in 50 infertile men with no defined cause and compared the SWE values to 100 testicles of 50 men forming a normal control group. The mean SWE measured values were 1.85 ± 0.31 m/s and 12.82 ± 5.19 kPa in infertile men, and 1.53 ± 0.25 m/s and 8.01 ± 3.02 kPa in the control group. Positive correlations were found between the testicular volume and SWE values in the infertile men group [63].

On the other hand, no clear differences in the SWE values between patients with varicocele, OAT, obstructive azoospermia, non-Klinefelter nonobstructive azoospermia or Klinefelter nonobstructive azoospermia were identified in a study conducted by Rocher et al. due to the substantial overlap in the values [49].

In 50 patients with nonobstructive azoospermia, Abdelaal et al. noted significant differences in SWE values between patients with successful and negative sperm retrieval using testicular sperm extraction (TESE). The SW values were 3.65 ± 1.24 kPa for positive sperm retrieval and 9.59 ± 6.68 kPa for negative sperm retrieval. Using a cut-off value of ≤4.125 kPa 2D SWE can be a predictive tool for negative sperm retrieval [64].

**Conclusion:** 2D SWE can be used to predict infertility but cannot differentiate between its cause because of the overlapping values. It can also predict successful sperm retrieval using TESE in men with nonobstructive azoospermia.

#### RTE

Using strain elastography for infertility assessment in patients, Küçükdurmaz et al. showed statistical differences in strain-ratio values between men with normal and abnormal semen analyses, with men with abnormal semen analyses having higher strain ratios + SR. Although FSH levels were statistically different in the two studied groups, there was no correlation between FSH values and the strain ratio [56].

When comparing obstructive and nonobstructive azoospermia in 1192 testes, Li et al. showed higher strain scores and strain ratios in nonobstructive azoospermia patients than in patients with obstructive azoospermia (median 0.490 vs. 0.340, *p* < 0.001) [65].

**Conclusion:** RTE can be used to differentiate between patients with normal and abnormal sperm counts. Furthermore, it can select patients that would benefit from deobstructive surgery or sperm retrieval.

### 3.2.8. Testicular Tumors

#### 2D SWE and RTE

**Figure 5:**
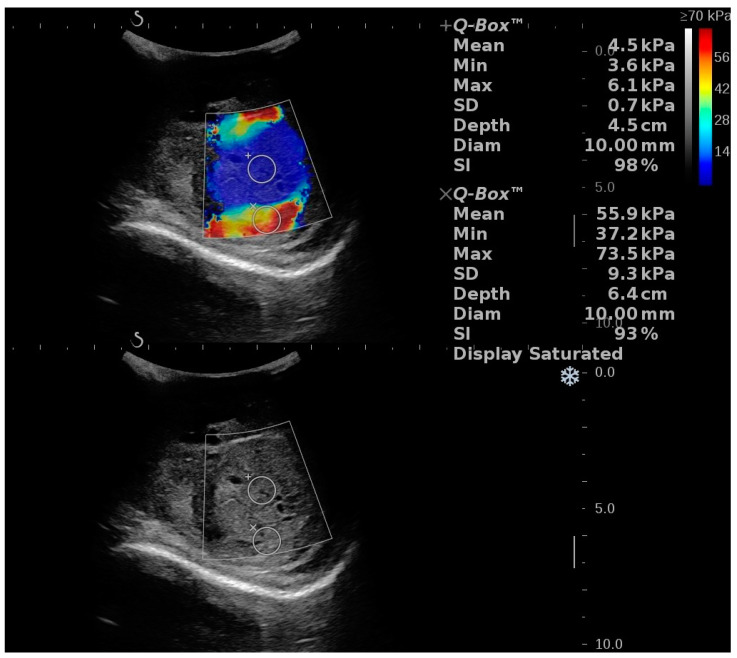
Illustration of a two-dimensional shear wave elastography (2D SWE) performed in a patient with a large testicular tumor using the SuperSonic Imagine Aixplorer Ultrasound device. A color scale map is displayed in the upper part of the image, low stiffness is color-coded with blue, while red signifies high stiffness. Numeric 2D SWE results (expressed in kPa) are displayed on the right side of the image. The Q-Box displays the mean, median, minimum, maximum and standard deviation (SD) of the measurements, along with the depth, the diameter of the region of interest (ROI), and the stability index (SI).

The use of elastography in the differential diagnosis of testicular masses has been reviewed by Fang C et al. showing promising results in differentiating benign vs. malignant testicular masses, with similar results for both RTE and 2D SWE [66]. A meta-analysis conducted by Lin et al. showed that malignant lesions were “stiffer” with both methods. However, an issue of overlapping results in these pathologies has been reported. The main causes of overlapping results were the presence of calcifications in benign lesions and necrosis/liquefaction in advanced malignant testicular masses [67]. Various degrees of stiffness can be obtained in large testicular tumors as seen in Figure 5. Different authors have attempted to differentiate seminomatous and non-seminomatous tumors using elastography for further investigation. The results are heterogeneous between studies and no clear indication can be deduced [68,69,70]. This heterogeneity could be attributed to the different ultrasound machines and probes used. Indeed, Shin et al. proved significant statistical differences between three machines used to measure SWE values on the same phantom probe [71].

**Conclusion**: Further studies using the T stage and tumoral size stratification are required due to the overlapping values in testicular masses. Due to the differences in probe production, different results may be obtained on the same type of pathology, thus making such examinations unreliable.

## 3.3. Penis

### 3.3.1. Normal Values

Zhang et al. examined the SWE values of corpus cavernosum in different sections in 40 healthy patients ranging from 19 to 81 years. The authors established similar values for corpus cavernosum stiffness for longitudinal and transverse sections (22.2 ± 63.5 vs. 22.2 ± 63.2 kPa, *p* = 0.817). They also found negative correlations between the SWE values and age and estradiol levels and positive correlations with testosterone levels [72]. Similar results regarding corpus cavernosum SWE values and their correlation with age were found by Inci et al. in a study conducted on 60 healthy volunteers [73].

**Conclusion:** There are no studies that evaluate the normal values of corpus cavernosum. In the case of 2D SWE, an increase in age and estradiol levels is correlated with a decrease in rigidity.

**Figure 6:**
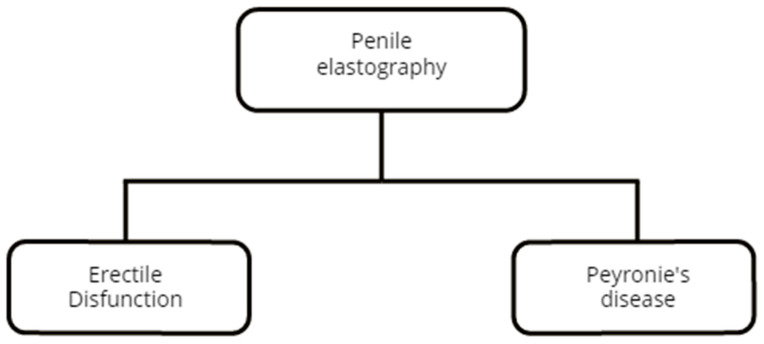
Penile pathologies evaluated by elastography.

The published usage of penile elastography is summarized in Figure 6.

### 3.3.2. Erectile Dysfunction (ED)

#### 2D SWE

*Examination in flaccid state*

Illiano et al. enrolled 270 patients and divided them into groups according to the International Index of Erectile Function (IIEF-5) score. SWE was performed with the penis in a flaccid state. The SWE values were higher as the grade of ED increased. Negative correlations between the SWE values and IIEF-5 and erectile hardness score (EHS) were found. A cut-off value of 24.75 kPa was found for ED prediction regardless of the cause [74].

Contradicting results were obtained by Turkay et al., which used SWE on 70 participants, including 35 patients diagnosed with erectile dysfunction (ED) with an IIEF-5 score of 17 and lower and 35 healthy volunteers. The examinations were performed with the penis in a flaccid state. The SWE values were significantly different in the two groups (20.94 ± 6.23 kPa and 24.63 ± 7.58 kPa, *p* = 0.027). A cut-off value of 17.1 kPa regarding the diagnosis of ED was established [75]. The limitation of the study, as addressed by the authors, was a lack of separation between the presumed cause of ED: hypertension (*n* = 10), diabetes mellitus (*n* = 20) and psychological (*n* = 5), a lack of histopathological confirmation and the use of a subjective quantification to assess ED.

*Examination in erect state*

Lee et al. compared SWEv of vascular, indeterminate and non-vascular ED patients after intracavernosal injection. There was a significant difference between the SWE values of arteriogenic, venogenic, indeterminate and non-vascular ED patients. The results indicated higher values in vascular patients with a cut-off value of 8.05 kPa for predicting vascular ED [76].

Cui et al. used SWE to compare rigidity changes of the corpus cavernosum in venogenic erectile dysfunction that was priorly diagnosed with Doppler US. There was no difference in SWE values between the studied groups for the flaccid state. Venogenic ED patients had significantly increased values after intracavernosal injection compared to non-vascular ED patients (13.73 ± 4.77 vs. 7.19 ± 2.09 kPa) [77].

Zhou et al. used SWE to differentiate arteriogenic vs. non-vascular ED after ICI (intracavernosal injection). They obtained a cut-off value of 7.75 kPa with a specificity, sensitivity, PPV, and NPV of 63.3%, 96.2%, 96.2%, and 70%, respectively [78].

Zhang et al. compared rigidity values of corpus cavernosum in patients with arteriogenic ED and non-vascular ED with healthy volunteers. Positive correlations between the flaccid and erect SWEv were shown. A cut-off value of 13.45 kPa with a specificity and sensitivity of 78.6 and 89.6% was used to differentiate arteriogenic ED patients vs. healthy volunteers in the flaccid state [79]. The authors concluded that SWE could be used to diagnose arterial ED.

Cheng H et al. studied the hardness of tunica albuginea and adjacent corpus cavernosum in patients with ED compared to healthy volunteers. The SWE measurements of the tunica albuginea were performed in the flaccid and progressive erectile states after two ICI of prostaglandin E1. SWE values significantly increased for each erection hardness (EH) grade as follows: resting state—21.66 ± 4.21 kPa; grade 1—32.61 ± 4.27; grade 2—54.86 ± 8.69; grade 3—128.02 ± 20.66; and grade 4—223.23 ± 23.61 kPa. Based on the EH grades, a cut-off value of 81.6 kPa was established as to whether sexual intercourse could be completed, since a grade 3 or 4 erectile hardness score is required for sexual intercourse completion [80].

However, it is debatable whether that which was measured as the tunica albuginea was the tunica itself, which is an anatomic structure with a thickness of around 0.9 mm. The sample was placed in a stiff area close to the transducer in the provided images, which anatomically contained part of the corpus cavernous, tunica albuginea, skin, and subcutaneous tissue. Furthermore, due to the close vicinity of the transducer and the sizeable mismatch of stiffness in that particular area, the recorded stiffness may not be entirely due to the tunica albuginea itself as it may also be altered by ultrasound shear wave transmission.

**Conclusion:** 2D SWE can differentiate arteriogenic, venous and non-vascular ED when used in combination with ICI. 2D SWE examinations with the penis in a flaccid state require further studies due to the contradicting results.

#### RTE

Altimbas evaluated the role of RTE on 88 patients with ED, with 57% having abnormal Doppler US findings. They used a semi-quantitative elasticity scoring system with lower scores attributed to softer tissues and higher scores to stiffer tissues. Compared to the normal group, the arterial failure group had the highest elasticity score (1.34 ± 0.17), while the borderline arterial failure and venous failure groups had lower scores (1.06 ± 0.83 and 1.03 ± 0.34, respectively) [81].

RTE was used to evaluate cavernous body fibrosis after retropubic radical prostatectomy. The patients were separated based on the nerve-sparing status—no, unilateral and bilateral nerve-sparing. Average elasticity scores were automatically given an absolute value between 0 (softest)and 5 (hardest) by the US machine while performing the elastographic examination. At both three and six months after the procedure, higher elasticity scores in favor of fibrosis were observed in the no nerve-sparing group compared to the other two. Higher elasticity scores showed a negative correlation with IIEF-5 scores [82].

**Conclusion:** RTE can be used to differentiate arteriogenic and venous failure ED. RTE can also be used to predict erectile function recovery after radical prostatectomy.

### 3.3.3. Peyronie’s Disease

In a case report, 2D SWE was used to identify the plaque and guide the intralesional injection treatment since B-Mode and Doppler ultrasonography showed no evidence of plaque [83].

RTE ultrasonography was used by Rivers et al. to evaluate 74 patients with complaints of Peyronie’s disease. While B-Mode ultrasonography identified penile plaques in 64 patients, RTE confirmed these findings and identified five more plaques in the ten remaining patients [84].

**Conclusion:** Sonoelastography can complement bidimensional and Doppler ultrasonography to evaluate, identify and guide treatment for penile plaques in the case of Peyronie’s Disease. Because only a small number of cases were reported, further studies are required.

## 3.4. Urethra

**Figure 7:**
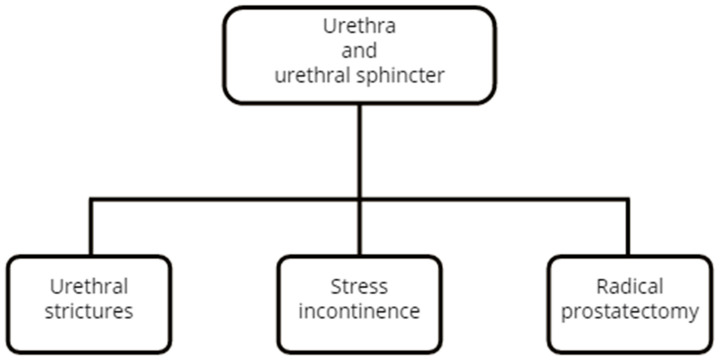
Pathologies of the urethra and urethral sphincter evaluated by elastography.

The published usage of elastography for urethra and urethral sphincter disease is summarized in Figure 7.

### 3.4.1. 2D SWE

Chung et al. used a combination of SWE and CEUS to evaluate patients with single bulbar strictures at the time of surgery and four months later. The measured stiffness of the spongiosum was greater at the site of the stricture (32.6 ± 5.4 vs. 27.3 ± 5.8 kPa) and in the case of narrower caliber strictures [85].

#### 3.4.2. RTE

Talreja et al. demonstrated that RTE has better diagnostic accuracy than sonourethrography and retrograde urethrography to estimate stricture location and length. RTE showed better accuracy for the depth of spongiofibrosis compared to sonourethrography (87.3% vs. 48%). RTE color scores were used. A significant association (*p* = 0.003) between the RTE color scores and the mucosa color on cystoscopy was found, whereas a non-significant correlation between the SE findings and the difficulty in incision was observed [86].

The same author used RTE to quantify the degree of spongiofibrosis in patients with urethral strictures. The color scores had a 100% concordance with severe fibrosis and an 87.5% concordance with a moderate degree of fibrosis. Strain-ratio values were significantly higher for histopathologically confirmed severe vs. moderate fibrosis cases (10.51 ± 2.297 vs. 6.33 ± 2.353) [87].

**Conclusion:** RTE sonoelastography seems to yield better accuracy than standardized examinations in the case of urethral strictures. The fact that sonoelastography is a non-invasive procedure in the case of this pathology can increase patient accessibility and compliance. No studies have been published on the use of pSWE. More studies are warranted on the potential use of 2D SWE in urethral strictures.

### 3.5. Urethral Sphincter

#### 3.5.1. 2D SWE Using the Transperineal Approach

It has been shown that SWE can be used to quantify the stiffness of striated urethral during different grades of contraction both in men and women. In both cases, the stiffness of the striated urinary sphincter increases with the intensity of the voluntary contraction [88,89]. Zhao B used supersonic shear wave imaging to compare the stiffness of the striated urinary sphincter in women with stress incontinence. Comparing 40 patients with stress urinary incontinence and 40 healthy subjects with no difference in age, as well as the body mass index and parity between the groups, they observed that the stiffness of the urethral rhabdosphincter muscle was significantly lower in women with stress urinary incontinence (mean values: 2.54 vs. 2.73 m/s; 19.7 vs. 22.7 kPa) [90].

#### 3.5.2. 2D SWE Using the Transrectal Approach

Tyloch et al. used transrectal ultrasound to evaluate the stiffness of the urethral sphincter in men after radical prostatectomy. They noted that a higher stiffness of the urethral complex correlates with better continence based on the International Consultation on Incontinence Questionnaire–Urinary Incontinence Short Form (ICIQ–UI SF) and the daily number of pads used [91].

**Conclusion:** 2D SWE may be used to evaluate the rigidity of the urethral sphincter in both women and men with urinary incontinence.

### 3.6. Urinary Bladder

**Figure 8:**
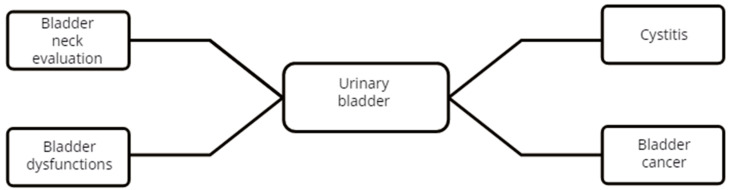
Urinary bladder pathologies assessed by elastography.

The published usage of urinary bladder elastography is summarized in Figure 8.

#### 3.6.1. Bladder Neck Evaluation

##### pSWE

Qian et al. assessed 36 female patients with bladder neck obstruction using a transperineal approach. They showed that the SWV values for the anterior and posterior lips were 2.44 ± 0.5 and 2.55 ± 0.68 m/s in the group with female bladder neck obstruction versus 2.02 ± 0.2 and 1.91 ± 0.23 m/s in the normal group. A SWV of 2.11 m/s for the anterior bladder neck lip and 2.06 m/s for the posterior lip was the best cut-off point for differentiating a bladder neck obstruction from a normal bladder neck [92].

##### 2D SWE

Sheyn et al. examined the bladder neck elasticity in 45 women with no urinary comorbidities using trans-abdominal SWE. The results yielded a median stiffness of 22 (17.1–28.2) kPa. An age above 45 years and a lack of vaginal deliveries were associated with higher SWE values [93].

##### 2D SWE vs. pSWE

Qian et al. used 2D SWE and pSWE using a transperineal approach to evaluate the bladder neck in 27 female patients diagnosed with bladder neck obstruction and 24 healthy females. Cystoscopy was considered the gold-standard evaluation method. The sensitivity and accuracy of pSWE were better than SWE in diagnosing female bladder neck obstruction. The combined use of pSWE and 2D SWE outperformed their separate use [94].

##### RTE

Using RTE, Ying et al. evaluated bladder neck compliance in 115 healthy women through a transvaginal approach. The strain ratio indicated that the anterior lip of the bladder neck was stiffer than the posterior one, with elastography indexes of 1.97 ± 1.08 and 1.32 ± 0.72. Age was the single independent factor correlated to bladder neck elasticity in this research [95].

**Conclusion:** In healthy patients, age is the common factor for a higher bladder neck outlet rigidity in women in all the studies mentioned above. Both 2D and pSWE can predict female bladder neck obstruction and their combined use yielded increased accuracy.

#### 3.6.2. Bladder Dysfunctions

##### 2D SWE

Sturm et al. used SWE to evaluate bladder pressure in children with bladder dysfunction. Based on the urodynamic assessment, the participants were classified as compliant and non-compliant. Measurements were taken in different filling stages. The SWE values correlated with bladder pressures, and the SWS measurements were higher in non-compliant bladders [96].

Do et al. used 2D SWE in 32 neurogenic bladder patients and showed a positive correlation between SWE values and intravesical pressure [97].

##### pSWE

As opposed to these results, Streur found that there was no significant correlation between bladder capacity and pSW speed measurements (*r* = −0.08, *p* = 0.7) or 2D shear wave speed measurements (*r* = −0.36, *p* = 0.09) in 25 children who underwent cystometrography [98].

**Conclusion:** Due to the contradicting results, further studies are required to establish the diagnostic value of SWE in the neurogenic bladder.

#### 3.6.3. Cystitis

Durmaz used 2D SWE to evaluate the bladder wall in the case of acute cystitis in 126 children. The SWE values were higher than in the healthy group. A cut-off value higher than 9.25 kPa yielded a sensitivity, specificity, positive predictive value, and negative predictive value of 92.1%, 88.1%, 89.3%, and 92.6%, respectively. Evaluations were performed independently of the bladder filling state [99].

**Conclusion:** Due to the high specificity, sensibility and independence of the bladder filling state, 2D SWE can be used as an alternative tool to diagnose acute uncomplicated cystitis in children when compliance to further examinations is low and urine collection sterility is questionable.

#### 3.6.4. Bladder Cancer

##### pSWE

Onur et al. used pSWE to examine the bladder tumoral masses in 39 patients presenting gross or microscopic hematuria. Transurethral resection of the bladder tumor was performed in all patients. They compared the stiffness of malignant and benign bladder masses and showed that malignant bladder masses were stiffer than benign ones (3.28 ± 0.74 m/s vs. 1.7 ± 0.82 m/s, *p* < 0.01). Transitional-cell-type masses showed increased SW values compared to squamous-cell carcinomas but with no significant difference (3.31 ± 0.79 vs. 3.04 ± 0.82 m/s). The patient pool had an unusually high number of squamous-cell carcinoma (25.64%) and nephrogenic adenoma (7.69%) when compared to the general incidence [100,101].

##### 2D SWE

Huang et al. used SWE to investigate the differences in the shear wave elasticity and collagen fiber content between low- and high-grade bladder urothelial carcinoma. High-grade carcinomas had significantly higher SWE values than low-grade carcinomas (13.35 ± 4.39 vs.8.73 ± 1.87 kPa) as well as higher collagen fiber areas (11.45 ± 1.66 vs. 7.64 ± 0.70%). There was a significant correlation between the SWE values and the collagen fiber area [102].

**Conclusion:** 2D SWE can be used as a preoperative tool to predict the level of differentiation of a newly discovered bladder tumor, thus guiding the urologist to a more profound and more extensive transurethral resection. At this state of knowledge, SWE cannot differentiate between TCC and squamous-cell carcinomas of the bladder. No studies have reported the usefulness of elastography in differentiating non-muscular vs. muscular invasive tumors.

**Final remark**: Ultrasound elastography has been commercially available for more than 15 years. It embraces a sizeable array of technical solutions. Despite the considerable number of applications in urology and publications, there is still a clear need for further work to define the role and proper methodology of the technique.
